# Mind the intention-behavior gap: a qualitative study of post-myocardial infarction patients’ beliefs and experiences with long-term supervised and self-monitored physical exercise

**DOI:** 10.1186/s13102-024-00987-2

**Published:** 2024-09-27

**Authors:** Alexander Svenningsen, Sylvia Söderström, Silvana Bucher Sandbakk, Lars Gullestad, Kaare Harald Bønaa, Ulrik Wisløff, Siri Marte Hollekim-Strand

**Affiliations:** 1 Faculty of Medicine and Health Sciences, Department of Circulation and Medical Imaging, Norwegian University of Science and Technology (NTNU), 8905, Trondheim, 7491 Norway; 2 Department of Neuromedicine and Movement Science, NTNU, Trondheim, Norway; 3 Department of Teacher Education, NTNU, Trondheim, Norway; 4 Institute of Clinical Medicine, Department of Cardiology, University of Oslo, Oslo, Norway; 5 Clinic for Heart Disease, St. Olav University Hospital, Trondheim, Norway; 6 Department of Circulation and Medical Imaging, NTNU, Trondheim, Norway; 7 Department of Neuromedicine and Movement Science, NTNU, Trondheim, Norway

**Keywords:** Exercise, Myocardial infarction, Behavior change, Secondary prevention, Cardiac rehabilitation

## Abstract

**Background:**

Many post-myocardial infarction (MI) patients struggle with physical activity behavior change (BC) for life-long secondary prevention. There is limited knowledge about factors influencing long-term physical activity BC among post-MI patients. This qualitative study aimed to explore the beliefs and experiences related to post-MI patients’ physical activity BC process following a year’s participation in a supervised and self-monitored exercise program: the Norwegian Trial of Physical Exercise After MI (NorEx).

**Methods:**

We conducted a qualitative study, performing in-depth semi-structured interviews with a randomly selected sample of NorEx participants when they were scheduled for cardiopulmonary exercise testing after one year of participation. Interviews were transcribed verbatim and the data was analyzed by applying reflexive thematic analysis.

**Results:**

Seventeen participants (*n* = 4 female [24%]; median age, 61 years; median time since index MI, 4 years) were recruited and interviewed once. Analysis resulted in four main themes (nine sub-themes): (1) Personal responsibility to exercise (Exercise is safe, Health benefits, Habitual exercise); (2) Peer social support for a sense of safety and belonging (Social exercise, Supervision is preferred); (3) Research participation transformed exercise beliefs (High-intensity exercise is superior, Personal Activity Intelligence (PAI) promotes exercise adherence); and (4) Mind the intention-behavior gap (Initial anxiety, Lack of continued follow-up).

**Conclusions:**

Several participants reported that they were able to maintain exercise BC during a year’s participation in NorEx. Nevertheless, a perceived lack of continued and individualized follow-up made some participants struggle with motivation and self-regulation, leading to an intention-behavior gap. Therefore, our findings suggest there is a need for individualized and continued social support and supervision from health and exercise professionals to maintain long-term exercise BC for secondary prevention among post-MI patients.

**Trial registration:**

The NorEx study has been registered at ClinicalTrials.gov (NCT04617639, registration date 2020-10-21).

**Supplementary Information:**

The online version contains supplementary material available at 10.1186/s13102-024-00987-2.

## Background

In all regions of the world, myocardial infarction (MI) is a leading cause of morbidity and mortality [1]. People with a history of MI are at increased risk of suffering recurrent major coronary events [2], making secondary prevention crucial [3, 4]. Epidemiological studies consistently find that physical activity is associated with a lower risk of all-cause mortality and cardiovascular morbidity [5, 6]. Patients who become more physically active after MI have a 50% lower four-year mortality risk compared to those who remain sedentary, and even small increases in regular physical activity are associated with 10–30% lower MI adverse event rates [7]. Consequently, physical activity has a Class I recommendation for secondary prevention and rehabilitation in European and US guidelines [8–10].

Traditionally, cardiac rehabilitation (CR) is divided into three phases: inpatient phase I CR, outpatient phase II CR, and phase III CR, the latter focusing on long-term secondary prevention and lifetime maintenance [11]. Strong evidence exists that supervised and exercise-based phase II CR programs, typically lasting 8–24 weeks [12, 13], lead to behavior change (BC) and increased cardiorespiratory fitness among patients with cardiovascular disease (CVD) [14]. However, when supervised phase II CR ends, many patients fail to maintain their newly acquired physical activity behavior. Moreover, CR referral rates have consistently been reported as low as approximately 20% [15], meaning that many patients never engage in organized outpatient exercise-based CR programs in the first place.

Differing explanations for the failure to change or maintain physical activity behavior among CR patients have been given [16, 17] and key barriers and facilitators among post-MI patients have been suggested. Social support by factors such as companionship and continuous physical activity supervision have been emphasized as important means to facilitate physical activity BC among Australian and British post-MI patients [18, 19]. Indeed, several qualitative studies have found that loss of continued supervision after phase II CR completion likely has a deteriorating impact on the patient’s physical activity BC process [18–20]. Thompson et al. [21] found that health benefits and the need to improve health are important motivations for physical exercise (henceforth termed exercise) among Northern Irish post-MI patients. Furthermore, many patients struggle with fear and anxiety after MI. Bäck et al. [22] explored Swedish post-MI patients’ perceptions of fear related to physical activity and exercise, showing that each patient’s dynamic coping process requires individualized person-centered strategies to increase participation.

Reviews of qualitative studies indicate that physical activity levels among individuals with CVD are influenced by a complex interplay between psychosocial factors, including beliefs, knowledge, and social support, as well as access to facilities [23]. Similarly, CVD patients’ perceptions of phase II CR participation seem to be shaped by an interplay of external (e.g., safety, accessibility, and social support networks), internal (e.g., fear, motivation, and mood), and cultural factors [24]. However, the reviews encompassing various CVD patient groups did not explicitly address experiences among post-MI patients. Overall, most previous qualitative studies were conducted among individuals with cardiovascular risk factors, or recent MI patients currently enrolled in phase II outpatient CR programs [25]. Consequently, there is a lack of knowledge of the factors influencing long-term exercise adherence among post-MI patients in phase III of CR.

Therefore, it is of interest to explore beliefs and experiences underlying an exercise BC process among post-MI patients who are engaged in phase III of CR. The Norwegian Trial of Physical Exercise After MI (NorEx) [26] provided the opportunity to carry out in-depth interviews with post-MI patients, exploring their unique experiences with long-term exercise adherence. Thus, the overall aim was to investigate the beliefs and experiences related to exercise among post-MI patients following a year’s participation in a supervised and self-monitored exercise program.

## Methods

### Study design

A qualitative study design was used to explore the beliefs and experiences expressed in the data. Semi-structured in-depth interviews were conducted, and reflexive thematic analysis [27] was applied for data analysis. At the time of the interview, the participants had been part of the exercise arm of the NorEx trial for one year.

### Setting

NorEx is an ongoing nationwide randomized controlled trial, determining the efficacy of 3.5 years of supervised home- and community-based exercise on mortality and cardiovascular morbidity in post-MI patients. Electronic Health (eHealth) tools are utilized with Personal Activity Intelligence (PAI) for self-monitoring and remote monitoring of exercise by study personnel. PAI is a personalized physical activity metric that considers the individual’s sex, age, and resting and maximal heart rate, and quantifies the weekly amount of physical activity necessary to reduce the estimated risk of cardiovascular morbidity and mortality based on continuous heart rate measurements [28, 29]. Intervention group participants are free to choose exercise modality, but frequency and duration should accumulate to at least 115 min of exercise weekly or a PAI score of 100, including a minimum of 20 min of high-intensity exercise (≥ 85% of peak heart rate) a week. Knowledgeable trainers, mainly physical therapists or exercise physiologists, are responsible for the supervised exercise intervention follow-up for groups of approximately 20–40 participants. Participants are invited to attend a 12-week introductory phase initiated by an educational workshop with their trainer, followed by four supervised group-based high-intensity interval training and strength training sessions with pre-defined educational content. Follow-up from trainers decreases after the in-person introductory phase, leaving participants to mainly exercise independently in their home environment with remote supervision via eHealth tools. Based on individual preferences and trainer opportunity, the following key BC strategies are applied in the continuation of the intervention: (1) Self-monitoring of behavior outcome (PAI); (2) Individual supervision, i.e., review of behavior goals and physical outcome; (3) Social support through involvement of a family or friend as co-participant, trainer, and/or exercise group initiatives; and (4) Customized eHealth platform to deliver the abovementioned strategies.

### Inclusion and exclusion criteria

To be eligible for participation in NorEx, all of the following inclusion criteria had to be met:

- Men and women who were hospitalized in a Norwegian hospital with an acute MI (Type I) during 2013–2022. Patients are included minimum three months after hospitalization when they are in a stable condition.
- Norwegian national identification number, able to communicate in Norwegian or other Scandinavian language, and not expected to emigrate during the study period.
- Age 18–79 years at the time when receiving study invitation.
- Being able to perform physical activity at an intensity level as prescribed for the intervention group, as determined by study personnel.
- Signed informed consent.

The following exclusion criteria could not be present at the time of enrolment:

- Participation in physical activity at a similar or higher intensity level than what is prescribed for the intervention group, as determined by study personnel.
- Participation or planned participation in endurance sport competitions.
- Cognitive impairment / dementia that may interfere with the participants ability to comply with the study protocol.
- Alcohol or drug abuse or serious psychiatric disease.
- Known CVD that may represent a contraindication for moderate or high-intensity physical activity, such as symptomatic valvular heart disease, a diagnose of obstructive hypertrophic cardiomyopathy, uncontrolled hypertension, in-compensated heart failure, serious arrythmia not under control after treatment, pulmonary hypertension, significant angina after revascularization and optimal drug treatment.
- Renal insufficiency requiring dialysis.
- Any end-stage somatic disease with short life expectancy or that is expected to interfere with the participants ability to comply with the study protocol, such as advanced cancer, chronic lung disease with exacerbations requiring hospitalizations, or other serious disease, as determined by study personnel.
- Inability to comply with the study protocol due to any physical disability, somatic disease, or mental problem, as determined by study personnel.
- Residing in a nursing home or other institution.
- Participating in another research study on physical activity.

### Recruitment

After one year of participating in NorEx, a random sample of participants from the intervention group in Trøndelag county (*n* = 35) was invited to undergo cardiopulmonary exercise testing. We used convenience sampling throughout the testing period, making intermittent phone calls to ask if participants were willing to additionally be interviewed and participate in the present study in conjunction with testing. None of the participants declined to be interviewed. Recruitment to our study continued until no new information was observed in the obtained data, implying that saturation of data was achieved [30]. No further interviews were conducted past this point.

### Semi-structured in-depth interviews

We used individual semi-structured in-depth interviews to obtain information about each participant’s unique beliefs and experiences with exercise. This method allowed collecting detailed descriptions of participants’ individual experiences through purposeful conversation, producing data from the interaction between the researcher and interviewees [31]. The interviews took place in an office adjacent to the exercise lab used in NorEx and were conducted by the first author, who had no existing relationship with the participants. We established an interview guide before the interviews (Table 1. Or see Appendix A for full interview guide). The interview guide was iteratively developed along the way based on participant responses in the initial interviews. Open-ended questions were mainly used, and the interview guide was used as a starting point and reference for follow-up questions throughout a naturally flowing conversation. All interviews were audio taped and transcribed verbatim.

**Table 1:** Excerpt of the interview guide with example questions from designated parts of the interview

**Part 1. Practical information and physical activity background**
- What was your activity level like and your relationship to physical activity when you were younger? - How did experiencing a myocardial infarction affect your physical activity pattern?
**Part 2. Discovering NorEx and the introductory phase**
- What expectations did you have to participating in NorEx? - Can you tell me about your experiences with the introductory phase of NorEx?
**Part 3. Status and plans for adherence**
- Now that you’ve been participating in NorEx for a while, how has it generally been going with exercise? - How is it going with making room for exercise in your everyday life after you started with NorEx?
**Part 4. Exploring barriers**,** facilitators and motivators**
- Some might feel anxious during exercise. How is that in your case? - As a part of NorEx you have the opportunity to use an app and a watch as support for exercise. How has it been using these?
**Part 5. Exercise beliefs and self-efficacy**
- Thinking about the time you’ve been part of NorEx; in what way have your beliefs towards physical activity changed? - How realistic is it that you’ll be able to continue with the amount of exercise you’re currently doing, for the next month? What about the rest of the study-period?

### Analysis

We chose reflexive thematic analysis for its flexibility [27], allowing constant switching back and forth between analytic phases. Furthermore, reflexive thematic analysis is suitable for an inductive approach to theme identification and focuses on developing themes that clearly capture the essence of central concepts in the data. Exploring a patient group’s experiences, it was important to apply a method capable of identifying patterns of meaning, with room for interpretation. The reflexivity in reflexive thematic analysis is a strength in openly discussing the influence of chosen analytical lenses. Our research group has backgrounds in clinical medicine, physical therapy, and exercise physiology, with extensive clinical and research experience ranging from various patient groups to elite athletes. Deep-rooted theoretical assumptions and previous involvement with NorEx and other participants from the same population have inevitably led to pre-conceived perspectives on beliefs and experiences with exercise among post-MI patients. This likely shaped the questions asked during interviews, the interpretation of data during analysis, and the resulting themes.

Reflexive thematic analysis and the six phases described by Braun & Clarke [27] were applied as a framework for the analysis to systematically, yet flexibly, identify patterns of meaning to develop themes from the data set. This rigorous and iterative process followed the following six phases: (1) Familiarizing with the data; (2) Generating codes; (3) Searching for initial themes; (4) Reviewing themes; (5) Defining and naming themes; and (6) Producing the report. To become familiar with the data, transcripts were read and re-read several times. Newly identified codes were added to a list of all identified codes, with a separate column containing adjacent data extracts in a spreadsheet (Microsoft Excel, Microsoft, Washington, USA). To gain perspective and enhance the identification of patterns, all codes were simultaneously added to a digital mind map (MindManager, Mindjet, San Francisco, USA) sorted by initial themes. When all transcripts had been coded and after several meetings refining the initial themes, the final themes were defined, named, and agreed upon by consensus of the research group. Group discussions were held to ensure that core concepts in the data would be clearly conveyed through agreed-upon themes. The step-by-step analytical process was mainly conducted by the first author A.S., with significant contributions from S.M.H., S.S., and S.B.S. All authors were involved in the discussion of the findings and producing the final report. The consolidated criteria for reporting qualitative research (COREQ) checklist was used for reporting the research findings [32].

### Ethical considerations

All participants were provided with information, written and verbal, about the aim of the study, data storage procedures, the voluntary nature of participation, and that they had the right to withdraw their consent at any time. Participants were assigned de-identified ID numbers to ensure confidentiality. Only members of the research group had access to participant data. Additionally, any personally identifiable information was removed from transcripts to ensure anonymity. Files from the tape recorder were encrypted with password protection and safely stored on a secure local university server. All participants gave written consent to participate. The NorEx study was approved by the Regional Committee for Medical Research Ethics (REK 2019/797), and this qualitative sub-study was approved by the Norwegian Center for Research Data (ref No. 865912).

## Results

In total, 17 participants (*n* = 4 female [24%]) were interviewed. The average duration of interviews was 52 min. The median age was 61 years (range 43–73 years) and the median time since index MI was four years (range 1–7 years) (Table 2). 53% of the participants had completed more than four years of higher education, 24% had less than four years of higher education and 24% had completed high school. Reflexive thematic analysis resulted in four main themes, encompassing nine sub-themes, which conveyed participants’ beliefs and experiences with long-term exercise after MI (Table 3).

**Table 2:** Participant characteristics

ID	Sex	Age (years)	Time since MI (years)	Education level	Employment	Civil Status
P1	M	62	3	> 4 years HE	Full-time	Married/Partner
P2	M	45	7	< 4 years HE	Full-time	Married/Partner
P3	M	64	4	HS	Full-time	Married/Partner
P4	M	59	7	< 4 years HE	Retired	Married/Partner
P5	M	60	3	> 4 years HE	Full-time	Married/Partner
P6	M	60	3	< 4 years HE	Full-time	Married/Partner
P7	M	61	7	> 4 years HE	Full-time	Married/Partner
P8	M	69	7	> 4 years HE	Part-time	Married/Partner
P9	M	73	6	> 4 years HE	Full-time	Married/Partner
P10	M	67	4	< 4 years HE	Retired	Married/Partner
P11	F	53	5	HS	Part-time	Married/Partner
P12	F	67	4	HS	Part-time	Married/Partner
P13	M	71	6	> 4 years HE	Part-time	Married/Partner
P14	M	53	5	> 4 years HE	Full-time	Married/Partner
P15	M	43	3	> 4 years HE	Full-time	Married/Partner
P16	F	68	1	> 4 years HE	Retired	Married/Partner
P17	F	51	1	HS	Part-time	Single

P = Participant, M = Male, F = Female, HE = Higher Education, HS = High School

**Table 3:** Overview of themes and sub-themes

Experiences and Beliefs Towards Exercise Post MI	
**Themes**	**Sub-themes**
Personal responsibility to exercise	Exercise is safe
	Health benefits
	Habitual exercise
Peer social support for a sense of safety and belonging	Social exercise
	Supervision is preferred
Research participation transformed exercise beliefs	High-Intensity exercise is superior
	PAI promotes exercise adherence
Mind the Intention-behavior gap	Initial anxiety
	Lack of continued follow-up

### Personal responsibility to exercise

Participants had different starting points regarding their experiences and exercise-related confidence. However, at the time of the interviews participants clearly believed that exercise was important to improve and maintain health and physical function. The participants viewed it as a personal responsibility to maintain health through habitual exercise, and they shared a persistent commitment to exercise. Several viewed MI as a life-changing event, driving them to embark on a BC trajectory towards becoming more physically active. For instance, Participant Six expressed the following:

It became a kind of turn-over when I got it (MI), then I had to try to be more physically active. I had to start exercising.

Most participants felt personally responsible for enhancing their health, performing exercise for longevity: to live as long as possible with optimal physical and mental function. Participant Five stated:

I exercise because it’s fun, not because I must. But if I must, then of course I want to exercise more. I value my life. So, I want to […] extend the part of life where I function properly.

This quote illustrates the motivation behind the feeling of responsibility; the desire to maintain physical function as long as possible. However, it was not only a responsibility to oneself, but also a familial responsibility to carry out the behavior. Family held deep emotional significance, and participants expressed that a primary motivation for exercising was to maintain their health for the sake of their loved ones, as mentioned by Participant 17:

Even though she’s all grown up and all, she (her daughter) still needs me sometimes. That is a motivational factor; to stay healthy for others and not just yourself.

### Exercise is safe

Although experiencing MI itself motivated exercise BC among several participants, others needed to be reassured and feel safe first in order to act on the felt responsibility to exercise. Participating in a supervised exercise program, either as part of phase II outpatient CR following acute MI or by participating in NorEx, convinced participants that exercise is safe. Nevertheless, supervision was still appreciated for a sense of safety. Participant Four expressed:

To exercise is completely harmless, you can exert yourself and go all out! Before I had the impression that you were supposed to take it easy.

The participant articulated the change in perception of exercise he went through, indicating a previous fear of exerting oneself that is no longer present. Thus, it appears that being convinced through education by health and exercise professionals, as well as experiencing exercise as harmless in practice, promoted continued exercise participation by securing a feeling of safety even with high-intensity exercise.

### Health benefits

When feeling safe enough to exercise with high intensity, several participants reported experiencing positive health benefits. Health parameters such as cardiorespiratory fitness, physical function, and mental well-being were perceived to have either been maintained or improved, as shown in the quotes by Participants Five and 13, respectively:

It’s been a long time since I’ve been in this good shape. I’ve never been in as good shape as after I had that infarction.

I feel like I’ve been getting in better shape, and I feel better both physically and mentally, so there’s no reason to not keep doing it and to maintain my fitness.

We found that these experienced health benefits led to feelings of increased competency, and thus enhanced motivation for continued exercise among the participants.

### Habitual exercise

Participants expressed a personal responsibility to exercise to improve and maintain their health and physical function. When feeling reassured about the safety of exercise and experiencing its physical and mental health benefits, many participants found it more manageable to maintain the new exercise behavior and making it habitual. Furthermore, setting behavior goals and establishing routines had also been important for sustained exercise BC, which Participant Seven described:

You must do things routinely. When I bike home, I routinely push pretty hard, and at the end I take a steep hill even though it takes me further away from home. I’ve made up this illusion that that’s where the road home goes, so it’s unacceptable to take a shortcut.

Several participants expressed that the first step of initiating exercise can be demanding, but once you can get going, it feels easier to continue. After having participated one year in the NorEx intervention program, several participants reported feeling an urge to get outdoors and be active, a feeling previously unheard of. This feeling was expressed by Participant 13:

It hasn’t been like this before, but now I feel that if several days go by and I haven’t been exercising in some way, then I feel like something is missing. I start to want to go outdoors and exercise.

Similarly, Participant 11 shared the following:

It feels good to see that it’s become one of those habits for me, where if several days go by and I’m not outside moving around then I feel the restlessness creep up on me.

### Peer social support for a sense of safety and belonging

Most participants reported that supervised exercise with peers provided social support and motivation, which facilitated the initiation, completion, and maintenance of exercise behavior. Participant Four expressed how the shared experience of having had an MI created a supportive environment for reflection and exchange of experiences:

I think it’s very positive to meet people who are in the same situation, to be instructed, to exercise, and to be gathered in a group. It’s literally a support group; yes, that’s the purpose it serves. I mean you feel safe when you meet up and share experiences.

The social support fostered a sense of belonging and commitment, thereby enhancing exercise adherence. Participants experienced group-based exercise as a means of providing and receiving feedback, positive reinforcement, and progress monitoring. Participant 17 said:

To be in that kind of group where there’s somebody who supports you no matter what because I’ve been the least fit person, but they still support and cheer me on when they fly past one round ahead of me. That support is there the whole time and I really appreciate it. And to notice, but also that the others notice that I’ve gotten in better shape.

Many valued the presence of peers who could motivate and push each other, whether directly through verbal praise or indirectly through social comparison.

### Social exercise

Although group exercise contributed to maintaining exercise behavior for the majority, participants still agreed that the social aspect of being physically active in groups is underestimated and should be emphasized to a greater degree. One example is the perspective of Participant Five:

For me, exercise is better when it’s social, and I haven’t gotten to know any of the 10–20 people that I’ve met at these gatherings. It could have been nice to get to know each other more and maybe exercise together but also do something else together.

Participants expressed a need to establish a social environment in the initial stages of group development, focusing on socialization rather than solely on exercise. They expressed that creating opportunities for social interaction could contribute to integrating exercise into participants’ daily lives and improve adherence beyond purely exercise-centered group sessions.

### Supervision is preferred

Many participants believed that group-based exercise sessions led by an exercise professional would enhance motivation, safety, and knowledge transfer. Participant 15 mentioned why supervision was important to him:

It’s something completely different when somebody’s there saying ‘Ok now this is going to happen, next session will be then, and only 30 seconds left!’ - that’s a different type of motivation in the exercise itself, I think. Because there are several others, we’re doing the same thing, and somebody is in charge.

Although many participants reported pushing themselves further in the presence of a trainer, some were content with non-supervised group sessions.

### Research participation transformed exercise beliefs

One year of participating in the NorEx intervention gave value to high-intensity exercise among participants. They found that the PAI metric incorporated in their wearable devices stimulated them to enhance their exercise intensity. Participant Five described how his beliefs related to exercise intensity had been altered:

And of course challenge the heart a little more, that’s something I’ve become more aware of based on what earns PAI and what doesn’t. That a two-hour walk only earns 1 PAI says everything. So, what I learned as a kid about how it’s not how fast you go, but how far that counts, that’s wrong. Completely wrong. At least when it comes to the heart. Doing some intervals on the other hand. I mean I don’t just walk up a hill, when I see a hill, I walk faster than I would have before.

### High-intensity exercise is superior

Participants strongly believed that high-intensity exercise is superior, improving cardiorespiratory fitness, cardiovascular health, and overall well-being, and yielding more PAI points, while lower-intensity exercise, recreational activities, and chores are inadequate for achieving similar outcomes. Therefore, most participants incorporated high-intensity exercise into their daily lives, either by aiming for an intensity of 85–95% of maximal heart rate during exercise or by simply adding a vigorous bout into daily activities. This was illustrated by Participant Five:

I’ve done nearly daily exercise by biking to work year around, and I throw in an exercise session on my way home. […] Towards the end I do a bout of maximal effort uphill biking for like 4–5 min. I go all out those last few minutes, and that’s something I do every day.

### PAI promotes exercise adherence

Furthermore, participants viewed the PAI scoring system as essential in order to maintain exercise behavior. Many participants identified achieving and keeping a weekly score of 100 PAI as their primary goal and motivator for exercise. This was the case for Participant 17:

I don’t think I’ll ever be super fond of exercising […]. I do, however, have a goal that I have to stay above 100 PAI every day. So today I really have to work hard, or my score is going to go way down tomorrow.

Some participants reported having established structured plans to maintain their PAI score, for others it simply provided extra motivation to go for a walk whenever a low PAI score was displayed. Participant 16 shared her experiences with exercise and self-monitoring using PAI:

It’s just like ‘Here’s the tool for you!’ I have a tool to do what I’m comfortable with. I think if we didn’t have this PAI thing where you can monitor yourself the whole time, then I think it would be hard.

Overall, the PAI scoring system provided motivation to exercise at higher exercise intensities. It served as a rewarding mechanism that kept them going, being described as both challenging and enjoyable at the same time. In general, participants clearly valued self-monitoring and found it motivating.

### Mind the intention-behavior gap

Although they were aware of the associated health benefits of exercise, some participants experienced ambivalence about exercise BC and sometimes struggled to translate their intentions into action. The participants listed several barriers to exercise BC, leading to an exercise intention-behavior gap. Participant 14 provided a fitting quote to illuminate the apparent intention-behavior gap:

[Talking about exercise] Sure, I think about doing it, but it’s like with Peer Gynt, ‘Think it, mean it, want it even, but to do it… no.’ That’s how it is.

### Initial anxiety

Participants expressed an initial fear of exertion and taxing the heart, which led to cautious physical activity and exercise immediately after MI, as explained by Participant Four:

I was a lot more cautious in the beginning, about how it would turn out, but now I’m a lot calmer and feel safe while I exercise.

For a few participants, experiencing MI also affected their mental well-being, taking a major toll on self-efficacy related to multiple aspects of life. Although most participants expressed that anxiety towards exercise diminished over time, Participant One and others remained vigilant for warning signs:

Sitting here now I don’t feel any anxiety for it anymore, but it’s still okay to keep an eye out.

### Lack of continued follow-up

When follow-up from trainers decreased after the introductory phase of NorEx, some participants explained how they had established exercise training routines and found organized group sessions unnecessary or simply preferred to exercise on their own. Conversely, some participants struggled to remain adequately physically active in their home environment. Thus, lack of continued follow-up impeded adherence to desired exercise behavior. Participant 14 was not satisfied with the follow-up he had received after the introductory phase of the intervention:

I have to say that for being in the group getting so-called follow-up, I don’t think there’s much difference between the follow-up and no follow-up groups. It’s important to establish routines and that takes more continuity than a few group-exercise gatherings in the beginning.

Some participants expressed that exercise intensity decreased when they exercised by themselves without observation. They wished they had somebody to push them harder than they were able to by themselves. Struggling with self-regulation, several participants expressed a need for more organized, frequent, and flexible supervised group-based exercise programs with peers of similar fitness levels and ages, as seen in comments from Participants Five and Six, respectively:

There could be more organized group sessions, a more stable program offered to us. So more big group sessions where you can just join when it suits you, maybe once a week.

People have very different starting points. You’re supposed to push yourself, and it’s difficult to push yourself when you’re exercising with somebody who’s at a completely different level.

While some expressed the need to interact and exercise in groups with peers, others highlighted the need for individually tailored exercise follow-up to fully encompass the unique situation of each individual and support the maintenance of exercise behavior.

Participants also expressed the need for more variation in training sessions to facilitate adherence, as participants found high-intensity interval training quite monotonous. Although high-intensity exercise was deemed valuable to most participants, some of the participants simply did not enjoy the exercise and several found high-intensity exercise especially physically and mentally challenging, as illustrated by Participant Four:

There’s no doubt that it’s pretty tough to complete if you’re supposed to do it x amount of times with somewhere between 85 and 95% effort. That sort of exercise is mentally tough to carry out if you’re going to do it by the book.

## Discussion

In this qualitative study, the reflexive thematic analysis resulted in four main themes: (1) Personal responsibility to exercise; (2) Peer social support for a sense of safety and belonging; (3) Research participation transformed exercise beliefs; and (4) Mind the intention-behavior gap.

### Personal responsibility to exercise

Several participants regarded experiencing MI as a life-changing event, turning exercise into a perceived necessity and personal responsibility to prevent recurrent events. This is in agreement with previous studies describing MI as a life-threatening wake-up call, triggering a desire to adopt positive lifestyle changes [18, 19, 21]. Thus, MI represents a momentum that could be harnessed for exercise BC, but is likely insufficient on its own to fuel long-term maintenance for most patients.

### Peer social support for a sense of safety and belonging

Furthermore, most participants in our study expressed a need for various social support to achieve long-term exercise BC, in particular peer social support by group-based exercise. This confirms previous qualitative research on MI and coronary artery disease patients emphasizing the value of social support as probably being one of the main factors influencing exercise BC [18, 19, 23–25]. Thus, social interaction should also be emphasized in phase III of CR.

### Research participation transformed exercise beliefs: high-intensity exercise is superior

To our knowledge, this was the first qualitative study conducted on post-MI patients currently participating in the exercise arm of a long-term randomized controlled trial. Thus, some findings reflected the novelty of this investigation. Participants valued and regarded high-intensity exercise as superior to lower exercise intensities or other physical activity. This may be due to the initial exercise-based educational program that emphasized the importance of exercise intensity to improve cardiorespiratory, and/or the PAI-metric that rewarded higher intensities. This raises the question of whether or not the belief of high-intensity superiority merely reflected social desirability bias with participants responding to meet the perceived expectations of the NorEx-researcher interviewing them. Nevertheless, our participants’ beliefs aligned with findings from a systematic review and meta-analysis by Qin et al. [33], which found that high-intensity interval training significantly improved exercise capacity in post-MI patients compared to moderate-intensity exercise and routine physical activity. Furthermore, participants’ beliefs aligned with previous qualitative studies on stroke patients in New Zealand [34] and Norwegian microvascular angina patients [35]. They also related the intensity of their efforts to the health gains made from exercise [34] and reflected on high-intensity exercise as being good for the heart [35], similar to the way our participants emphasized the importance of sufficiently challenging the heart to improve cardiovascular health. This indicates that engagement in a long-term, supervised, and self-monitored exercise program may impact health literacy and empower post-MI patients in their choice of exercise mode and intensity.

Many of our participants regularly carried out the high-intensity exercise program for a year, even though they did not find it particularly enjoyable. This contradicts a previous study suggesting exercise enjoyment is key for long-term exercise adherence among post-MI patients [21]. However, our participants clearly identified with expected health-related outcomes of high-intensity exercise, implying that the meaningfulness of the activity played an important role in adherence. A review of self-determination theory and exercise underscored the importance of fostering both intrinsic motivation, such as enjoyment of the activity itself and identification with the outcome in order to promote BC [36]. Thus, the noted lack of enjoyment implies a need for personalized follow-up in ongoing interventions to reduce the risk of non-adherence.

### Research participation transformed exercise beliefs: PAI promotes exercise adherence

Self-monitoring and feedback by the PAI-metric were expressed as a primary motivating factor by participants in our study. PAI provides a reward in terms of heart rate-based points given when physically active above a certain threshold. While higher intensity yields more points, points are withdrawn with inactivity over the course of a week. This may serve as a form of external regulation of motivation, in accordance with self-determination theory [37].

Our participants were particularly motivated by the intrinsic nature of the weekly goal to maintain 100 PAI. Similar to our participants, Australian patients in phase III of CR (33% post-MI) described PAI as motivating and showed significantly increased physical activity levels after a six-week intervention [38]. Furthermore, feasibility studies on type 2 diabetes patients in the US and Australia found that 80% were satisfied with PAI and intended to continue using PAI after the 12-week interventions [39, 40].

### Mind the intention-behavior gap: lack of continued follow-up

Even though PAI was utilized and found motivating as an individualized approach to exercise prescription, participants expressed a need for more personalized follow-up. Some participants struggled to reach their weekly PAI goal, and lack of variety in their exercise program may have contributed to the perceived intention-behavior gap. Similar to our participants, post-MI patients in the study of Coull and Pugh [19] requested more tailored physical activity advice to meet individual needs. Overall, individually tailored approaches seem integral to improving exercise adherence after coronary events because the motivation for exercise participation varies from patient to patient [41]. This emphasizes the importance of motivational communication from knowledgeable professionals, incorporating personalized behavioral goal-setting and regular evaluations to ensure variation and enjoyment in the exercise BC process to reduce non-adherence over time among post-MI patients. However, CR programs generally seem to lack variation and individualization [19].

Furthermore, randomized controlled trials are seldom systematically planned according to BC constructs, such as variation in motivation between and within individuals, or interventions are poorly described in terms of BC components [42]. Individual readiness to change behavior may be explained by the transtheoretical model of BC [43]. For instance, individuals aware of exercise benefits but not regularly physically active are typically characterized as being in the contemplation or preparation stage, whereas those engaged in an exercise program are typically in the action or maintenance stage. At the time of the interviews, most of our participants were likely in the latter stages. However, the cycling of stages is a normal and expected part of the non-linear BC process described with the transtheoretical model of BC [44]. Thus, we could expect that our participants may have progressed or relapsed, possibly both, during their first year of participation. Moreover, people can be expected to remain in the maintenance stage for six months to five years [43] and possibly even an indeterminate period [44]. This also underscores the importance of continuous follow-up and individualization for long-term exercise behavior maintenance in phase III CR. According to Prochaska & Velicer [43], optimal programs for health promotion should apply proactive, interactive, and stage-matched interventions. NorEx does not implement the latter, which could negatively impact long-term adherence due to a lack of individual stage-targeted BC strategies.

Phase III CR guidelines direct health practitioners to provide patients with advice regarding independent maintenance of long-term BC [8–12]. However, according to our findings and previous qualitative studies on post-MI patients [18–20], advice for independent maintenance is not sufficient to achieve lasting exercise BC. Thus, existing guidelines under-emphasize the importance of implementing continued, individualized, and person-centered follow-up to facilitate independent physical activity behavior maintenance in phase III of CR. Furthermore, current practice is unclear about who bears the responsibility for continued follow-up. To address these necessities, solutions must be feasible, realistic, and sustainable within the future healthcare system.

A possible strategy could involve integrating health services and fitness industries to oversee continued follow-up during phase III CR. Integration would rely on leveraging knowledgeable professionals within the field of exercise as medicine with profound insight into the mechanisms of life-long lifestyle BC.

Another possible solution could be to use information technology in long-term CR delivery. Studies have shown promising results in terms of the feasibility of teleCR [45, 46]. However, there is a lack of evidence for the long-term benefits of digital interventions to increase physical activity in people with chronic diseases [47]. A review by Gold et al. [48] showed that digital interventions may enhance physical activity behavior in healthy adults for three-to-six months, but the effects diminished after 12 months. Findings from our study suggest that eHealth interventions with sporadic individual follow-up in post-MI patients may not be sufficient for long-term maintenance. Perhaps a solution could be to combine self-monitoring devices with regular contact with knowledgeable professionals. A randomized controlled trial by Van Hoye et al. [49] on physically inactive healthy adults suggests that such combinations could maintain physical activity participation; however, the intervention only lasted four weeks followed by assessments after three, six, and 12 months. Results from our study can offer valuable input to improve exercise behavior maintenance in planning future randomized controlled trials, especially when considering the aspect of BC, which is inevitably present in long-term secondary prevention trials.

### Strengths and limitations

We used convenience sampling, recruiting interviewees from the ongoing quantitative NorEx study. Since interviews were conducted on participants having volunteered for an exercise intervention, selection bias may have occurred because our interviewees were likely more motivated for physical activity BC to begin with and not fully representative of the post-MI population. Our results may also be limited by potential recall bias, particularly where participants were recalling perceptions and experiences from the early stages of the NorEx intervention. Additionally, interviews were conducted after one year of NorEx participation during the Covid-19 pandemic and participants’ experiences with exercise may have been affected by restrictions at the time. However, applying reflexive thematic analysis and its reflexive core provided strength to the analysis of interviews in terms of openness about the research group’s theoretical backgrounds, the setting of the study, and the authors’ prior involvement in NorEx and similar projects. On account of the explorative and qualitative nature of our study, our findings are not representative of the entire post-MI population but may be transferable to other post-MI patients in similar contexts.

### Implications

Researchers and health professionals designing and implementing future randomized controlled trials or other secondary prevention interventions for post-MI patients should place greater emphasis on evidence-based strategies to assist individual BC processes. We advise implementing core concepts from the field of BC theory, such as the transtheoretical model of BC to tailor dynamic and stage-matched interventions for post-MI patients according to individual motivation, characteristics, and preferences.

The importance of establishing a social environment, not solely focusing on exercise, as well as customizing exercise groups by characteristics such as age and fitness level, should be considered for post-MI patients to promote long-term exercise adherence. Simultaneously, it is equally necessary to ensure patient-centered individualized follow-up by applying tools such as motivational communication, behavioral goal setting, and reviewing. Follow-up from health and exercise professionals should optimally be formalized and not discontinued in phase III CR, even when eHealth solutions are utilized. Exploring resource-efficient alternatives, such as combinations of individualized and supervised exercise programs and innovative digital interventions, should be considered. There is still a need for more research on exercise BC to assess the feasibility and effectiveness of secondary prevention strategies beyond phase II CR.

## Conclusions

Our findings contribute to the evidence of factors influencing long-term exercise adherence among post-MI patients in phase III of CR. High-intensity exercise was experienced as valuable and superior to lower exercise intensities to improve cardiorespiratory fitness and cardiovascular health. Using eHealth tools to self-monitor exercise with PAI was a major motivating factor for exercise adherence, and social support remains important. Nevertheless, a perceived lack of continued and individualized follow-up made some participants struggle with motivation and self-regulation, leading to an intention-behavior gap. Therefore, our findings suggest there is a need for individualized and continued support from health and exercise professionals in phase III CR to maintain long-term exercise BC for secondary prevention among post-MI patients.

## Electronic supplementary material

Below is the link to the electronic supplementary material.

Supplementary Material 1

## Data Availability

The data used and/or analyzed during the current study are available from the corresponding author on reasonable request.

## Abbreviations

MI: Myocardial infarction
CR: Cardiac rehabilitation
CVD: Cardiovascular disease
BC: Behavior change
NorEx: The Norwegian Trial of Physical Exercise After MI
PAI: Personal Activity Intelligence
eHealth: Electronic health
