# Supplementary material for: Mind the intention-behavior gap: a qualitative study of post-myocardial infarction patients’ beliefs and experiences with long-term supervised and self-monitored physical exercise
Source: BMC Sports Sci Med Rehabil. 2024 Sep 27;16:204. doi: 10.1186/s13102-024-00987-2 (PMC11437989; doi:10.1186/s13102-024-00987-2)
Supplement: Supplementary file 1 — Supplementary Material 1 [file 13102_2024_987_MOESM1_ESM.pdf]

	**Introduction:** Before the interview and audio recording begins, the information in the information sheet is repeated, and the informant is assured of voluntariness, anonymization, and confidentiality.<br>**Informants:** Participants in NorEx (region: Trøndelag)<br>**Objective:** Obtain information about the experience of changing and maintaining physical activity level through participation in NorEx (12 months after starting).	
	**Interview:**	
	Information about the purpose of the interview:	Participant's experience of participating in NorEx - with a focus on factors that promote or inhibit changes in physical activity levels and exercise.
	Information about the interview situation:	<u>Repeat information from the information sheet:</u><br>Voluntariness/Consent, confidentiality, anonymization of data, communication, publication, audio recording, possible to withdraw at any time, the opportunity to not answer questions if desired.<br><br>Provide a summary of the topic areas we will address.
	**Questions**	**Follow-up questions**
	**Part 1:**<br>**Practical information and physical activity background**	
1	Tell me about yourself:<br>• Age<br>• Gender<br>• Marital status<br>• Occupational status	
2	What was your activity level like and your relationship to physical activity when you were younger?	• Was your family active? *(to get an idea of activity level)*<br>• What impact do you think this has had on how you have experienced physical activity/exercise later in life?
3	Have you previously tried to change your exercise habits? *(Before joining NorEx)*	• How did it go?<br>• What made you succeed?<br>• *Alternatively:* What caused you to revert to old habits?<br>• *Alternatively:* What experiences did you gain back then that you brought into your NorEx participation?

4	How did experiencing a myocardial infarction affect your physical activity pattern?	• *If changed as a result of the event:* What has enabled you to achieve this?<br>• *If not changed but changed intention:* What could be the reasons for not actually increasing physical activity/exercise?<br>• *If neither intention nor change made:* What could be the reasons for this?<br>• Is it different now than it was then? Has this changed over time, from when you first had the myocardial infarction until now?
	**Part 2:**<br>**Discovering NorEx and the introductory phase**	
	About background and expectations for participation in NorEx	
6	When you heard about NorEx, what was the main reason you wanted to participate?	• Can you tell a little more about that? *(to get an idea if they had to consider it or not, why?)*<br>• Did you decide to participate on your own? Or were you recommended to join?
7	What expectations did you have to participating in NorEx?	• In what ways has NorEx met/not met your expectations?
	**About the introductory phase**	
8	Can you tell me about your experiences with the introductory phase of NorEx?	• What did you learn in the initial phase?<br>• How much did you feel you were able to influence your own exercise plan?<br>• How long did it take before you felt you were really getting started with the exercise/change? Why do you think it turned out that way?
9	Have you had a specific action plan for your exercise?	• *If made a plan:* How has this worked for you?<br>• *If no plan:* How has this worked for you (conscious choice?) *(Reasons why it worked/didn't work)*
	**Part 3:**<br>**Status and plans for adherence**	
10	Now that you've been participating in NorEx for a while, how has it generally been going with exercise?	• What are the reasons you succeed?<br>*Alternatively:*<br>• What are the reasons you don't succeed?<br><br>• Are you satisfied with your physical activity level?<br>• Would you like something to be different in this regard?<br>• To what extent do you experience joy with the exercise you do?

11	During this period, have you had any short or longer breaks from the change (exercise) you have done in connection with NorEx participation?	• What was the reason?<br>• What did you do then?<br>• How did you manage it?/what do you think is the reason it was difficult?<br>• *Given that you have changed exercise habits with NorEx:* Do you have a plan for what to do if you were to fall back into old habits?
12	How is it going with making room for exercise in your everyday life after you started with NorEx?	• Why do you think you succeed/don't succeed?
13	To what extent do you feel you have personal control over your exercise routine?	• Why do you think it's like that?<br>• Do you ever feel guilty, for example, for not exercising?<br>• What happens if you feel you don't want to exercise? (strategies to start exercising again?)
	**Part 4:**<br>**Exploring barriers, facilitators and motivators**	
14	Some might feel anxious during exercise. How is that in your case?	• *If anxiety:* What needs do you have in that regard? Could NorEx have helped with that?<br>• *If no anxiety:* Reflect, good to hear that you feel safe in exercise situations.
15	Some people feel prevented from exercising by physical pains, or concern about getting pains in the body from exercising. How is it for you?	• Why do you think it's like that? *(that you don't have problems with this or that you have problems with it)*
16	Some people feel that the effects of the medications they use prevent them from exercising. How is it for you?	• *If problems:* What have you done to get help/advice on this?
17<br><br><br><br><br><br><br>18	Some people feel they need more knowledge about exercise and their body to be able to continue with the changes you have made. How is it for you?<br><br>Some people feel they need to be sure they can handle the exercise they are going to do before they get started - How is it for you?	• Why do you think it's like that for you?

19	Have you chosen to have an exercise buddy with you, and how does that work?	• What do you think is the reason it works well/not so well?<br>• How important is it for you to exercise with someone?
20	How do you experience that friends and family support your exercise?	• Can you give examples?<br>• Have there been others who have contributed to you succeeding/not succeeding in changing your activity level?
21	How do you feel that your physical surroundings (your local area) are adapted for you to exercise and be physically active?	• Why do you think it's like that? What could be changed so that you were more likely to succeed/what is important for you to succeed?<br>• What opportunities do you have around you to do be as physically active as you have planned to be?
22	As a part of NorEx you have the opportunity to use an app and a watch as support for exercise. How has it been using these?	• Why do you think it's like that for you?<br>• What makes you succeed/not succeed?<br>• How could things have been done differently to make it even better for you?
23	How does the PAI point system work for you?	
	**Experiences with NorEx trainer**	
24<br><br>25	What has your coach meant to you during the period you have participated in NorEx?<br><br>How do you experience your relationship with your NorEx coach?	• Why do you think it's like that? - reasons for good/not so good?<br>• What do you think the NorEx coach could have done differently?
26<br><br>27	Are there any other things you can think of - that we haven't talked about so far – that prevent you or make it difficult to change your exercise habits?<br><br>Are there any other things you can think of - that we haven't talked about so far - that help you change your exercise habits?	

	**Part 5:**<br>**Exercise beliefs and self-efficacy**	
28<br><br><br><br><br><br>29	*If made changes:* How do the changes make you feel?/How have the changes you have made affected what you do in your everyday life?<br><br>*If made changes and experienced progress:* What do you think is the reason for that?<br>*If no experience of progress:* How do you think you can achieve a sense of progress?	• Are you more active and participate more, for example? Do you have more energy/feel that you can endure more?
30<br><br><br><br><br><br><br><br>31	*If made changes:* How long do you think you will continue with the exercise you are doing now? *If no changes made:* To what extent do you think it is possible for you to make a lasting change in your physical activity level in the future?<br><br>*If made changes:* How realistic is it that you'll be able to continue with the amount of exercise you're currently doing, for the next month? What about the rest of the study-period?<br><br>*If no changes made:* How realistic do you think it is that you will start exercising in the next few months?	• What is your plan going forward?<br>• *If you have an action plan:* What is your action plan for the next two-three months?<br>• Reasons why you think it's realistic/unrealistic?
32	*If made changes:* To what extent would you say that exercise has become a habit?	• *If a habit:* what do you think you have done to make it work?<br>• *If not a habit:* What do you think is the reason for that?
33	Thinking about the time you've been part of NorEx; in what way have your beliefs towards physical activity changed?	• What do you think is the reason for that?

	**Part 6: Summary and conclusion**
	• Is there anything else you can think of that would improve your experience of being a NorEx participant?<br><br>**• Summary** *(summarize what we have talked about based on the main points)*<br><br>• Is there anything that hasn't been mentioned that may be relevant to what we've talked about now - or that is important for your exercise going forward? / Is there anything else you can think of that may be important that we haven't talked about?<br><br>• Can I contact you to clarify or confirm if there is any uncertainty regarding your answers during this interview?<br><br>• Is there anything you would like to ask me before we conclude the interview?<br><br>Thank you very much for participating in this interview.
